# A new perspective to understand public response to the Typhoon Doksuri from coastal and inland regions

**DOI:** 10.1016/j.heliyon.2024.e36862

**Published:** 2024-08-24

**Authors:** Mengqiong Xu, Juanle Wang, Zheng Qu, Xiaodong Min, Yamin Sun

**Affiliations:** a School of Marine Technology and Geomatics, Jiangsu Ocean University, Lianyungang, 222005, China; b State Key Laboratory of Resources and Environmental Information System, Institute of Geographic Sciences and Natural Resources Research, Chinese Academy of Sciences, Beijing, 100101, China; c Jiangsu Centre for Collaborative Innovation in Geographical Information Resource Development and Application, Nanjing, 210023, China; d School of Civil and Architectural Engineering, Shandong University of Technology, Zibo, 255049, China; e School of Resources and Environment, Institute of Disaster Prevention, Sanhe, 065201, China

**Keywords:** Typhoon disaster, Data mining, Disaster prevention and mitigation, Public opinion

## Abstract

Massive amounts of data from social media possess the potential to rapidly identify the primary issues of concern in emergency disaster management. In summer 2023, Super Typhoon Doksuri which was an exceptionally special typhoon disaster that caused severe damage to China's coastal areas and disastrous impacts in inland regions, particularly triggered the most severe rainstorm in Beijing area in over a century. To enhance typhoon hazard reduction in both coastal and interior locations, it is crucial to examine public response to these events. This study uses microblog text data from July 27 to August 3 of 2023 to map the public response to Typhoon Doksuri. The Support Vector Machine (SVM) algorithm was used to classify the microblog text in combination with the typhoon path to analyze the spatial and temporal variations of the emotions of the affected individuals. The relationship between changes in public opinion, the distribution of topics, and the major disasters triggered by the residual circulation of Typhoon Doksuri in the Beijing-Tianjin-Hebei region is discussed. The Mentougou mega-storm in Beijing area that occurred in July 2023 is a typical case. The findings demonstrate that during the typhoon event, the focus of public attention changed with the movement of the typhoon path, and various public opinion topics exhibited temporal synchronization. Public sentiment indicates that the overall supportive sentiment is higher than is fearful sentiment. Based on this, it is crucial to strengthen the Beijing-Tianjin-Hebei cooperative emergency response, and response measures were proposed related to urban flood control and drainage construction, public awareness, backward areas, secondary disasters, resident relocation, and social media technology.

## Highlights

• Using the Support Vector Machine method to classify microblog topics, a high accuracy of up to 92.83 % was obtained.
• Multi-level categorization and identification of public emotions , categorized into a maximum of 21 categories.
• Case study of extremely heavy rainfall in Beijing, exploring the response differences between coastal and inland areas.

## 1 Introduction

**Fig. 1:**
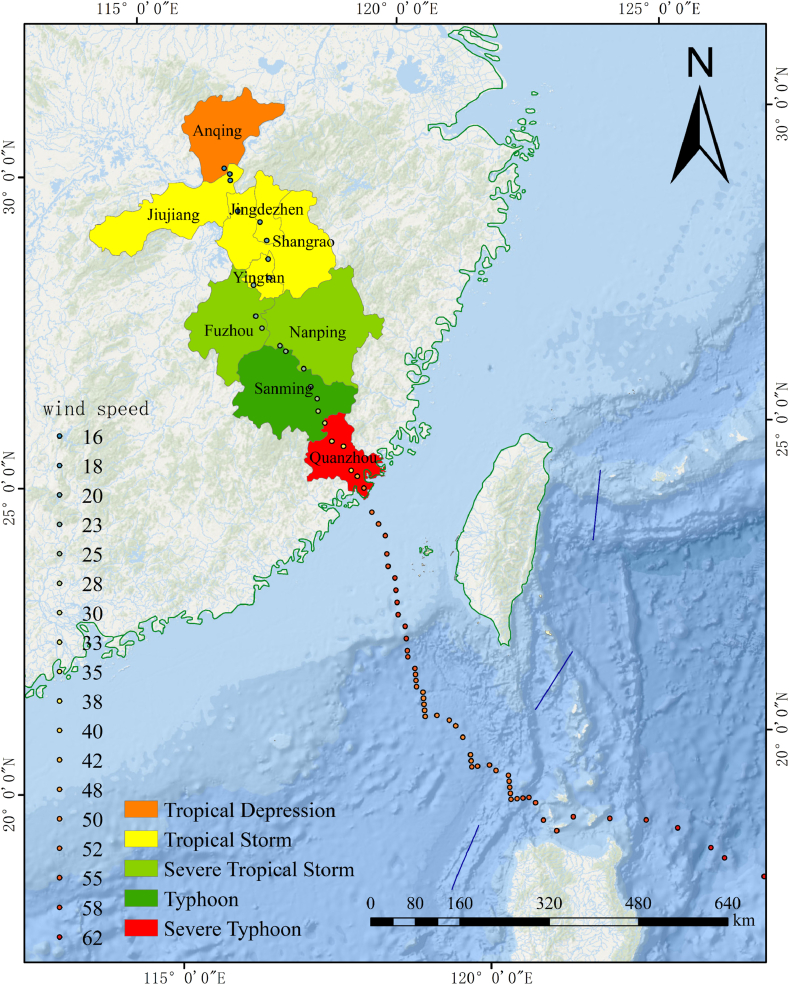
Track and intensity change of Typhoon Doksuri.

Major natural catastrophes have occurred more frequently in recent years due to climate change and extreme weather, thus making it difficult for individuals to survive safely and maintain societal stability. An average of seven tropical cyclones make landfall in China each year, thus making China one of the nations with the most frequent typhoon storm surges that cause catastrophic damage [1]. Between 2013 and 2022, China experienced 95 typhoon storm surge processes and 66 typhoon storm surge catastrophes. These events resulted in many dead and missing individuals as well as approximately 6800 million yuan in direct economic losses [2]. 2023, No. 5 super Typhoon Doksuri caused widespread devastation, remarkably, it followed an uncommon occurrence for its direct landfall in Fujian without traversing Taiwan, maintained its super typhoon intensity for over 70 h, impacting both China and the Philippines severely. Fig. 1 illustrates the path of movement and intensity changes of the typhoon. Following its cessation, residual circulation continued northward, penetrating deep inland and triggering extreme rainfall in northern China. That resulted in 2.91 million individuals being affected to varying degrees in Fujian, Zhejiang, Anhui, and Jiangxi and 768,000 individuals being relocated to emergency shelters [3]. It caused Beijing rainstorms and other major disasters at the end of July and resulted in a total of 63 deaths in the Beijing-Tianjin-Hebei region and 34 individuals missing. Typhoon disaster tracking, disaster aid, and disaster evaluation can all benefit from timely mastery of social media data mining. The public can use social media platforms to express their opinion on disasters, emergency assistance, regional disaster situations, personal emotions, and other disaster-related topics. Official governments can use these platforms to release weather warnings, emergency responses, and other messages when unexpected disasters occur. Big data from social media have also progressively emerged as a new data source for monitoring catastrophe-related events [4].

In recent years, many scholars have utilized social media data such as that from Twitter and Sina Weibo to conduct topic identification, sentiment analysis, and disaster dynamics monitoring. Many algorithms can be applied to text categorization. Two stem extraction methods were proposed by Rianto et al. [5], with a text classifier model being established for the categorization of 550 Indonesian language instances. Accuracy rates of 73 % and 85 % were achieved, respectively. Wahid et al. [6] annotated the text using Latent Dirichlet Allocation (LDA) for topic modeling, employed Bidirectional Encoder Representation from Transformers (BERT) for text embedding, and utilized deep learning classifiers for the categorization of disaster datasets. A comparison with alternative methodologies revealed that this approach achieved the highest accuracy, reaching 84.1 %. Bruijn et al. [7] extracted hydrological features from a global precipitation dataset and aggregated them onto a multimodal neural network, through which flood-related tweets on Twitter were classified, achieving a precision of 91 %. A short text classification method based on convolutional neural network (CNN) and semantic extension was proposed by Wang et al. [8], aiming to classify six different text datasets. An accuracy of up to 91.34 % was achieved. In the study conducted by Londo et al. [9], news articles in the Indonesian language were classified using SVM, Multinomial Naive Bayes, and DT (Decision Trees). It was found that the performance of the SVM algorithm was superior, with an accuracy reaching 93 %.

Sentiment analysis focuses on categorizing texts into positive, negative, and neutral. According to Ragini et al. [10], emotions were categorized into 3 classes using machine learning methods and into 2 classes (positive and negative) based on lexicon lists. It was found that the lexicon-based approach was more suitable for emotional analysis of the public during disasters. Saddam et al. [11] categorized community sentiments during Jakarta Flood into 3 categories, positive, negative, and neutral, based on SVM and found that negative sentiments far outnumbered positive sentiments. In a study conducted by Lee et al. [12], text about the Sewol Ferry disaster on YouTube, Twitter, and Facebook platforms was analyzed, also found that negative sentiments on all three platforms significantly outweighed positive sentiments. Yang et al. [13] employed a CNN model to categorize tweets during the Ya'an earthquake into 6 classes. It was found that a prevalence of fear and anxiety emotions characterized the early stages, whereas positive sentiments came to predominate in later stages. Gruebner et al. [14] proposed a semantic modeling approach for extracting 7 negative emotions during the Sandy hurricane period to assess public post-disaster mental health, revealing that sadness and disgust were the most prominent emotions. Karmegan et al. [15] utilized the emotional lexicon to partition the emotions expressed in Twitter tweets before, during, and after the 2015 food shortage disaster in the Chenna region of India into 8 categories, and found incidence of negative emotions was highest across various regions.

The focus of typhoon disaster social media data analysis is generally concentrated on large-scale analyses spanning national or even global scopes, or along coastal regions. Boas et al. [16] analyzed the social media responses of citizens in Xiamen, China during the landfall of Typhoon Meranti and found that Xiamen citizens valued official information on social media platforms more. During the Sandy hurricane period, geographical geotagged Twitter data generated from coastal counties affected in New York, New Jersey, and Connecticut were selected by Pourebrahim et al. [17] for analysis, revealing that the fluctuations in these tweets were consistent with the varying stages of the disaster. Zhang et al. [18] analyzed topic changes and sentiment differences during the period when central Philippines were hit by Typhoon Haiyan. It was found that the number of tweets, population, and the proportion of young and middle-aged population are the predominant factors contributing to the differences in emotional space. Using social media data regarding the 2013 Super Typhoon Haiyan, Shen et al. [19] explored worldwide online social responses and found that global online social responses are not significantly correlated with geographical distance and vary with the development of disasters.

Among numerous text classification models, it has been demonstrated by previous research that the classification performance of SVM algorithm is positioned at a comparatively elevated level [9,20,21]. SVM is suitable for short text classification with high-dimensional sparse features, and it performs better in small sample datasets, such as microblog tweets [22,23]. So the SVM model is selected in this study for text topic classification, and the comprehensive performance of the classification results reaches up to 92.83 %. Previous sentiment analyses, whether based on machine learning or sentiment dictionaries, have mainly categorized sentiments into 3 categories: positive, negative, and neutral, with only a few performing secondary categorization. The sentiment lexicon was used to identify sentiments in disaster-related texts and categorize them at multiple levels, i.e., Level 1, Level 2, and Level 3. Ultimately, the tertiary emotional classification yielded 21 distinct categories. Previous most studies focus mainly on the coastal areas and neglect other areas. This could indicate that social media data were not fully utilized, and this could lead to a one-sided public opinion analysis. Based on this, this study analyzes social media data during the impact of Typhoon Doksuri and provides in-depth analysis of the Mentougou mega-storm event that occurred in July 2023. We perceived the public opinion dynamics and the emotional characteristics of microblogging users at different stages during the disaster. We also propose measures for future disaster responses with the intention of providing support for emergency relief and mitigation work during typhoon disasters.

## 2 Materials and methods

### 2.1 Research data

Typhoon Doksuri was listed as one of the top ten natural disasters in China in 2023 [24] and one of the top ten weather and climate events in China in 2023 [25]. It was the most powerful typhoon to make landfall on the Chinese mainland in 2023 and the second-strongest typhoon to hit Fujian since 1949. Microblog texts were collected within the timeframe from July 27, 2023 (the day before Doksuri departed from the Philippines and reached landfall in China) to August 3, 2023 (when the remnants of the Doksuri circulation had completely dissipated). The official API of the Sina Weibo Data Center was used to conduct an advanced search using the keyword “Typhoon Doksuri”. The content of each text encompasses various attributes, including username, user location, text, creation time, attitudes_count, comments_count, reposts_count, and other associated attributes. A total of 91,432 records were collected for this study.

Data preprocessing was performed to remove irrelevant information from the massive text data. This was primarily processed using regular expressions to remove spaces, retweets, emoticons, links, and replies from other microblogs. The corpus was subdivided using the Jieba lexical tool and combined with the customized user lexicon to subdivide the corpus of microblog text, and there were a total of 89,187 available microblog text data. The details of the dataset are presented in Table 1.

**Table 1:** Summary description of the microblogging dataset.

Attribute Information	Value
Total amount of data	89187
Average text length	140.81
Standard deviation of text length	150.40
Minimum value of text length	2
Maximum value of text length	4642
Time frame for data collection	2023-07-27 to 2023-08-03
Language	Chinese

### 2.2 Method

The SVM is a supervised machine learning algorithm proposed by Corinna et al. [26] that can be trained to obtain support vectors. For text categorization, given a set of training samples A = { (x_1_,y_1_),(x_2_,y_2_),(x_3_,y_3_), …,(x_n_,y_n_)},y_i_ ϵ {-1,+1}, the sample categories were obtained by testing training set A, and the test set was then classified based on the obtained categories. The SVM seeks an optimal classification hyperplane in the sample space that meets the classification requirements based on the training sample set A to maximize the blank area on both sides of the hyperplane. A 30 % portion of the sample data were set aside for testing, while 70 % were used for training. The SVM model was then created and used to classify all text data from the microblogs. SVM can utilize kernel functions to transform input features into a higher-dimensional space. This transformation enables initially linearly non-separable data to become linearly separable in the higher-dimensional space. As shown in Equation (1), the classification rule function of SVM can be expressed as:

(1) $$F\left(x\right)=\text{sign}\left(\sum_{i=1}^{n}c_{i}a_{i}^{*}K\left(d_{i}，d\right)+b\right)$$

$c_{i}$ is the text category; $a_{i}^{*}$ is the Lagrange multiplier; $d_{i}$ is the training text; d is the text to be classified.

This study focused on the sentiment analysis of microblog texts based on the Sentiment Vocabulary Ontology Database provided by the Dalian University of Technology Information Retrieval Laboratory (DUTIR). The emotional classification system of this ontology library is constructed based on Ekman's six primary categories of emotions. However, the DUTIR database is not universal, and we therefore combined the characteristics of Typhoon Doksuri to expand the content of the Sentiment Vocabulary Database.

The evaluation indexes include Precision, Recall, and the comprehensive evaluation index F1 value. These evaluation indexes were used to calculate the Precision(Equation (2)), Recall(Equation (3)), and F1(Equation (4)) for each topic category as well as for the secondary and tertiary emotion categories separately. The calculation formula is as follows:

(2) $$\text{Precision}=\frac{\text{Num}\_\text{correct}}{\text{Num}\_\text{extraction}}$$

(3) $$\text{Recall}=\frac{\text{Num}\_\text{correct}}{\text{Num}\_\text{person}}$$

(4) $$F_{1}=\frac{2*\text{Precision}*\text{Recall}}{\text{Precision}+\text{Recall}}$$

Num_correct denotes the number of tweets where the calculated category result matches the manual labeling; Num_extraction denotes the number of tweets determined to be in that category as a result of the calculation; Num_percision denotes the number of tweets manually labeled as being in that category.

## 3 Results

### 3.1 Typhoon disaster text

#### 3.1.1 Word frequency of disaster

**Fig. 2:**
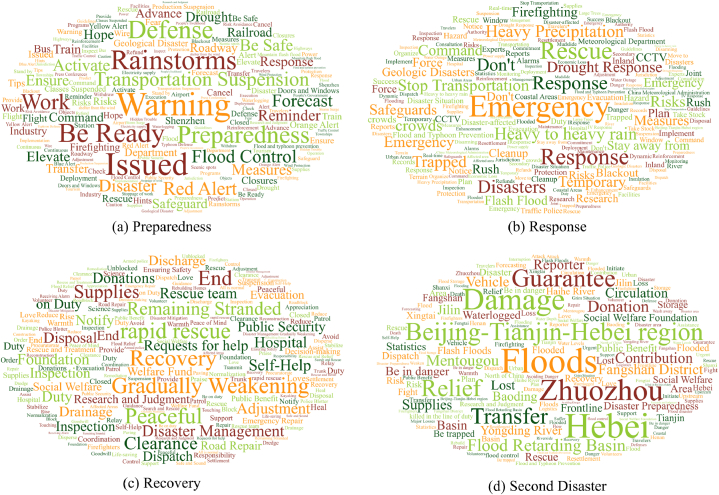
Changes in microblog word clouds during the impact of Typhoon Doksuri ((a): Preparedness, (b): Response, (c): Recovery, (d): Second Disaster).

Fig. 2 presents the shifts in public attention at various points over time. In preparedness (Fig. 2(a)) as indicated, words related to disaster warning such as “Warning”, “Be Ready”, “Defense”, and “Rainstorms” appeared more frequently, thus reflecting the high alertness of the public to Typhoon Doksuri. During the response phase (Fig. 2(b)), as the typhoon made landfall disaster information such as “Stop Transportation”, “Risks”, and “Safeguards” were widely mentioned, and words such as “Emergency”, “Response”, “Rescue”, “Measures”, and “Command” became the focus of public discussion. Subsequently, the typhoon became a tropical storm, the wind speed slowed down, and the focus of public attention gradually changed to “Recovery”, “Supplies”, “Gradually Weaking”, “Clearance”, “Hospital”, and other post-disaster recovery information (Fig. 2(c)). Since then, Typhoon Doksuri had dissipated, but its residual circulation was still northward rippling through northern China with the Beijing-Tianjin-Hebei region as the primary affected area, and the words “Beijing-Tianjin-Hebei region”, “Zhuozhou”, “Floods”, and “Flood Retarding Basin” became the focus of public attention (Fig. 2(d)). The word frequency was visualized in a manner that corresponded to the features of the modifications to the Typhoon Doksuri disaster scenario. Specifically, its high-frequency vocabulary shows different discussion tendencies as the typhoon's path changes and the extent of damage caused varies.

#### 3.1.2 Overall and disaster area topic identification

The primary topics during Typhoon Doksuri were categorized into five types (Table 2) that included Typhoon Trajectory (topic 1), Alert Notifications (topic 2), Emergency Response (topic 3), Rescue and Relief (topic 4), and Disaster Information (topic 5). Generally, in response to the disaster situation, the typhoon event caused huge disaster losses, and the public discussion primarily focused on “Disaster Information” that accounted for 45.8 % of the discussion. This was followed by “Alert Notifications” that accounted for 15.8 % and “Emergency Response”, “Typhoon Trajectory”, and “Rescue and Relief” that accounted for 14.8 %, 12.4 %, and 11.2 %, respectively. The results indicate that during a disaster, for different topics, the attention of the public exhibits obvious distribution characteristics, particularly in areas of advanced perception in the pre-disaster period and disaster information after the disaster occurs, and the public experiences a higher level of concern.

**Table 2:** Microblog topic discussion.

No.	Categories	Topic Description	Percentage
Topic 1	Typhoon Trajectory	Typhoon, Track, Landfall, Kilometers, Center, Wind-force, Sea level, Westbound, Distance …	12.4 %
Topic 2	Alert Notifications	Warning, Be Safe, Alert, Issue, Signal, Alarm Precaution, Tip, Reminder …	15.8 %
Topic 3	Emergency Response	Deployment, Emergency, Measures, Research, Reinforcement, Exhaustion, Closure, Organize, Against …	14.8 %
Topic 4	Rescue and Relief	Alarm, Firefighting, Rescue, Soldiers, Foundation, Supplies, Punchbowl, Donations …	11.2 %
Topic 5	Disaster Information	Late, Shut down, Canceled, Collapsed, Flash floods, Ferocious, Waterlogged, water outage, power outage …	45.8 %

**Fig. 3:**
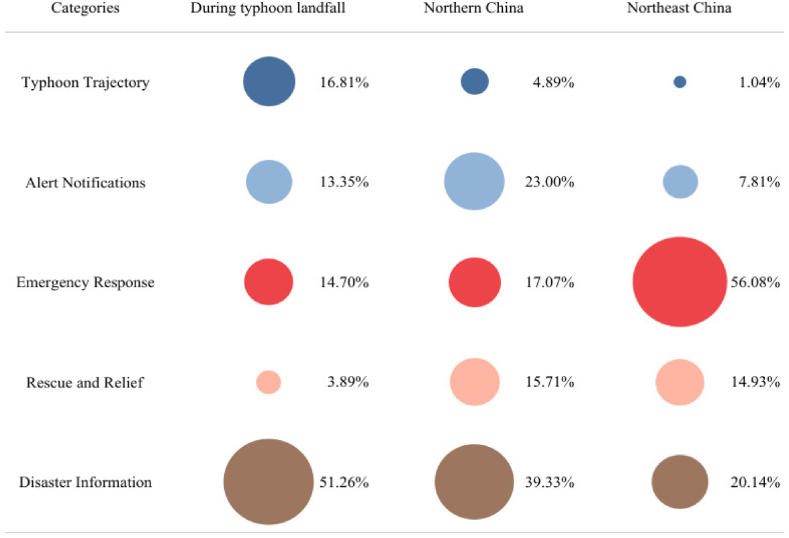
Topic categories in disaster areas at different stages.

According to the trajectory changes of typhoon paths and its residual circulation extending from coastal to inland areas, three major regions affected by disasters were selected within different time frames to investigate the categories and variations of public's topics (Fig. 3). The first disaster area is the region affected from the beginning of Typhoon Doksuri's impact on China to the time when the typhoon's numbering was stopped; the second region represents the North China region, with the Beijing-Tianjin-Hebei area as the representative, affected by the residual circulation of Typhoon Doksuri, and the third region comprises the Northeast China region affected by both the residual circulation of Typhoon Doksuri and the impact of Typhoon Khanun.

Overall, the proportions of public topics at each stage are generally consistent with the overall sentiment topics, with a higher proportion focused on topics such as “Disaster Information” and “Rescue and Relief”. Concerning individual topics, public interest in the “Typhoon Trajectory” shows a gradually declining trend, initially characterized by fervent discussions owing to abundant official forecast information, which subsequently diminishes following the cessation of typhoon designation. The “Alert Notifications” exhibits a trend of initially increasing and then decreasing. Gradual rise due to typhoon landfall and northward influence of residual circulation in the first period, and gradual weakening of residual circulation in the later period, which becomes less powerful and the hotness of discussion is weakened. The public's attention to the topic of “Rescue and Relief” has been gradually increasing. Less attention was paid to the lack of preparedness in the early stages of the disaster. When the residual circulation affected the Beijing-Tianjin-Hebei region, it was discussed more because of the improved emergency response capacity. As the residual circulation weakens and enters the Northeast, the level of public discussion of “Rescue and Relief” slightly decreased, but there still remained a high level. Reflects the fact that as the duration and severity of the disaster changed, the focus of public attention gradually shifted from the disaster itself to the disaster prevention and mitigation measures and the results of disaster relief.

Changes in public topics are highly correlated with the depth of the typhoon's path, the transformation of the affected area, and the local emergency response capacity. Regardless of the stage, the local public is generallyand consistently more concerned about official responsiveness, and the disaster prevention and relief measures for the disaster. These concerns reflect not only the public's expectations for safety, but also their high level of concern about the performance of the government and the community in responding to disasters.

### 3.2 Spatiotemporal trends in typhoon disaster topics

#### 3.2.1 Time series of topics for typhoon disaster events

**Fig. 4:**
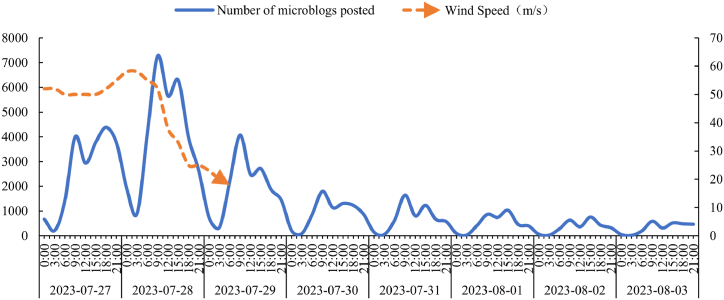
The curve of number of microblog tweets over time, July 27, 2023–August 3, 2023.

The time series of microblog topics during the impact of Typhoon Doksuri on China was delineated into 3-h intervals as illustrated in Fig. 4. On July 28, 2023, at 9:55 a.m. Typhoon Doksuri made landfall in China. The number of microblog topics related to Typhoon Doksuri peaked at 9:00 a.m. on the same day. Every day, the microblog subject conversations reached their climax at approximately 9:00 a.m., after which they progressively became less heated. Notably, the volume of microblogging on the Sina Weibo platform was quite low, the public became active between 6 and 9 a.m., after 9 a.m., interest in social media subjects decreased. However, topic discussions peaked again at around 12:00 or 18:00, this is in line with the daily behavioral patterns of the public. Furthermore, we can also find when the typhoon's wind power reaches its peak, with more people concern this disaster event, and the number of tweets also hits its highest consequently, and when the wind power weakens, the number of tweets gradually decreases. Statistical tests were conducted for this purpose, and the results (Table 3) show a strong relationship between the number of microblogs posted and wind speed, with a correlation coefficient of 0.89 and showing significance at the 0.01 level.

**Table 3:** Correlation between number of microblogs posted and wind speed.

		Number of microblogs posted
Wind Speed	Pearson's R	0.89**
	p-value	0.003
	R^2^	0.77

*p < 0.05 **p < 0.01.

The time series of Typhoon Doksuri exhibited rapid eruption and swift ascension that was followed by a gradual decline and a protracted recessionary phase. Before the typhoon made landfall, microblog subject discussions were high, it peaked on the day that the typhoon landed and then gradually diminished, shows a massive outbreak in a short period of time. After stop numbering, its residual circulation move northward, ultimately triggering extreme rainstorms in northern China. The public continues its topic discussion and pays attention to the impact and response to secondary disasters, so its microblog topic opinion possesses characteristics of a protracted recession.

**Fig. 5:**
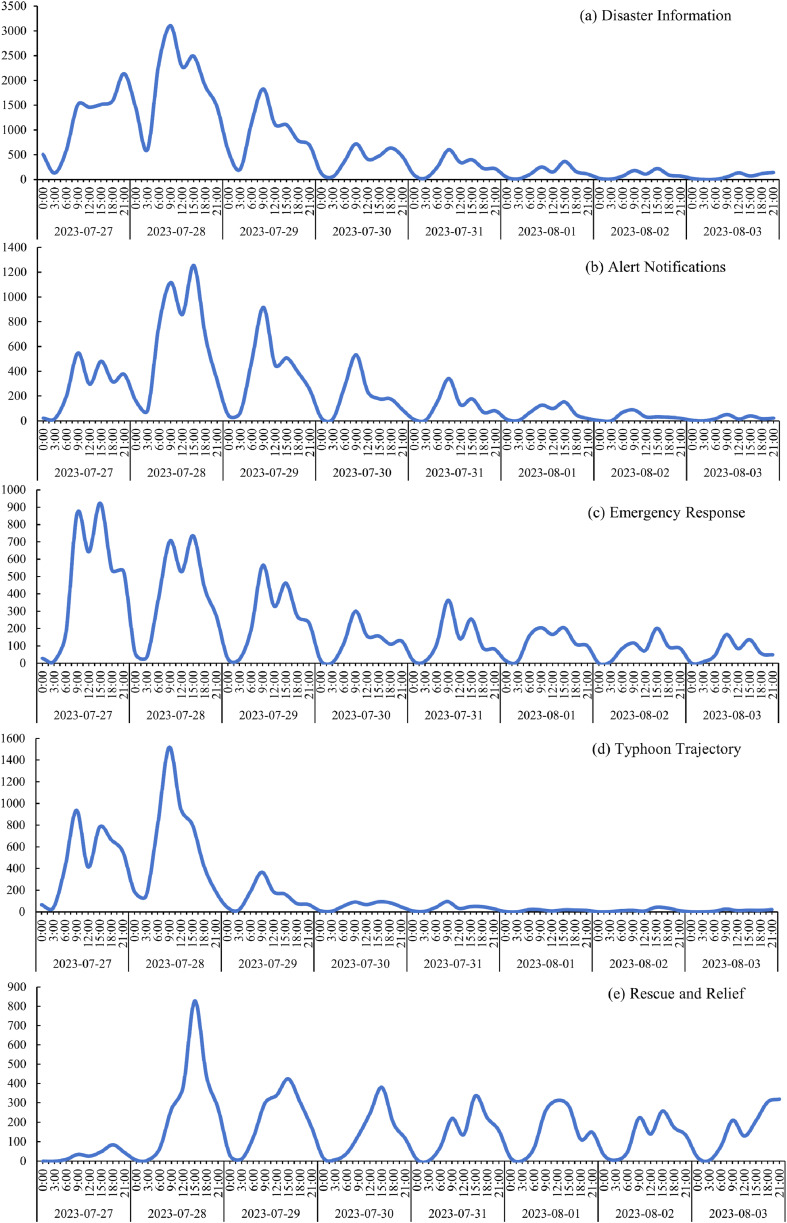
Curve of microblog topics over time, July 27, 2023–August 3, 2023 ((a): Disaster Information, (b): Alert Notifications, (c): Emergency Response, (d): Typhoon Trajectory, (e): Rescue and Relief).

Fig. 5 shows the attention of the public was primarily focused on microblogging topics the day before and on the day of the typhoon landfall, and the public paid more attention to information regarding the impacts of the disaster on China as well as the damage and other information regarding the situation. Additionally, on the day before typhoon landfall, there was a greater degree of attention focused on the “Emergency Response” (Fig. 5(c)) message, thus indicating that reflects timely implementation of response measures by both official and the public. After the departure of the typhoon, topics such as “Disaster Information” (Fig. 5(a)), “Alert Notifications” (Fig. 5(b)), “Emergency Response”, and “Rescue and Relief” (Fig. 5(e)) exhibited small fluctuations, the discussion of “Typhoon Trajectory” (Fig. 5(d)) stagnated, and this is in concordance with the disaster characteristics of Typhoon. The fluctuations in the discussion of “Rescue and Relief” messages were the most pronounced, thus reflecting the high level of public concern regarding secondary disasters caused by the northward movement of the residual circulation of “Doksuri”. There was a greater inclination among the public at this time to focus on how the official government and rescue teams were executing and progressing their relief and rescue efforts amid the intense secondary disaster scenario. This reflects the public's expectations and concerns regarding disaster response and rescue efforts.

The attention of the public towards Typhoon “Doksuri” demonstrates varying trends, which are influenced by the occurrence, progression, and cessation of the disaster. In future disaster response, more attention should be paid to the timely release and dissemination of rescue information to meet public demand and improve the efficiency of disaster response.

#### 3.2.2 Spatial pattern of topics in typhoon disaster events

**Fig. 6:**
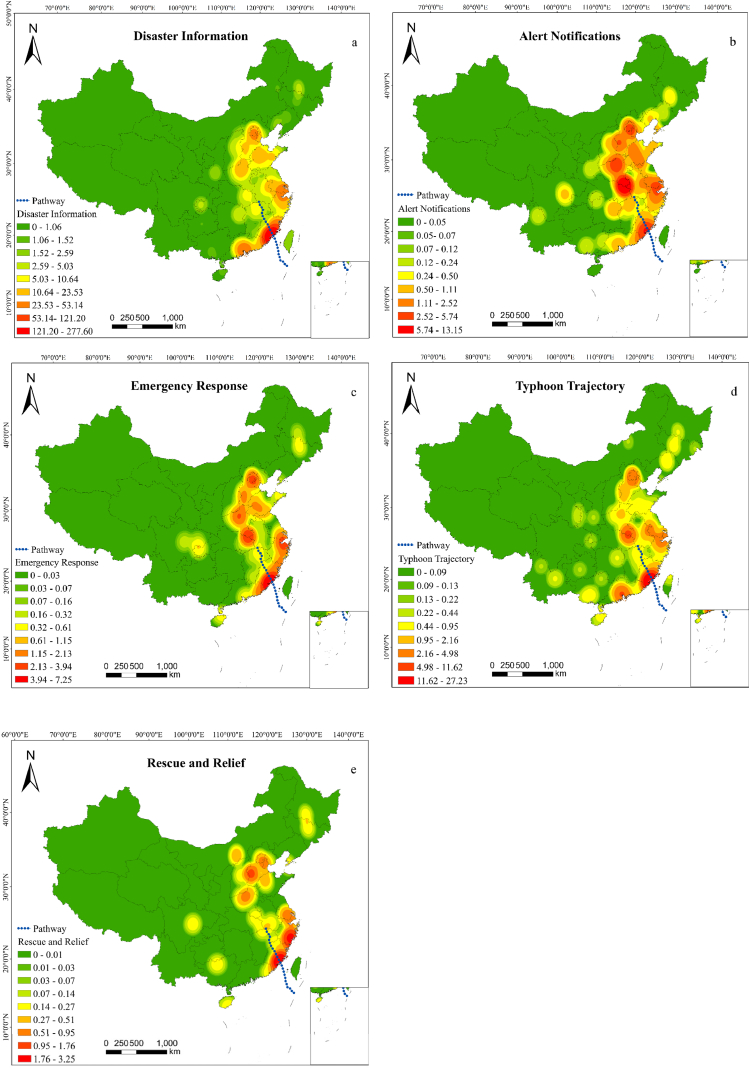
Spatial responses to microblog topics, July 27, 2023–August 3, 2023 (a: Disaster Information, b: Alert Notifications, c: Emergency Response, d: Typhoon Trajectory, e: Rescue and Relief).

Geostatistical analysis methods were employed to filter the microblog data with geolocations in the Chinese region. Next, the kernel density of the check-in data was analyzed for each topic category in the microblog text categorization (Fig. 6). Weibo discussions along the typhoon's track gradually waned. Fujian Province, where the typhoon made landfall, was the most frequently discussed area, whereas Anqing, Anhui Province, was less discussed as the typhoon weakened. Residual circulation northward, ultimately causing a major disaster in the Beijing-Tianjin-Hebei region. This can be observed as a significant increase in the discussion of microblog topics centered on the region, reflecting that changes in public opinion of typhoon disasters are in line with the trend of the lifecycle. The “Typhoon Trajectory” was discussed most intensely in the vicinity of the typhoon landing and departure sites and with less intensity in other regions. Subjects such as “Emergency Response”, “Disaster Information”, and “Rescue and Relief” exhibit spatial kernel density distributions centered around the features of the typhoon. They exhibited noticeably high kernel density values at the landing locations and comparatively low values in transit areas, then rose in the Beijing-Tianjin-Hebei region. Regarding “Alert Notifications”, the information was widely disseminated across cities that may be affected during the typhoon landing and residual circulation, thus indicating swift governmental responses to disaster warnings and meteorological information dissemination and an efficient disaster-prevention awareness among the public in various regions.

Additionally, a correlation was noted between the level of conversation among microblogging subjects and the economic progress of the region. The regions exhibiting higher kernel density values as presented in Fig. 6 are primarily located in the coastal region, the Beijing-Tianjin-Hebei region, and the central region that are more densely populated and economically developed, whereas the western region exhibits lower kernel density values and lower discussion intensity, as it is far from the impact area of the disaster and possesses relatively average economic development. These findings further demonstrate the close relationship among the hotness of microblog topic discussions, geographic location, and level of economic development.

### 3.3 Multi-layered emotions in typhoon disaster

**Fig. 7:**
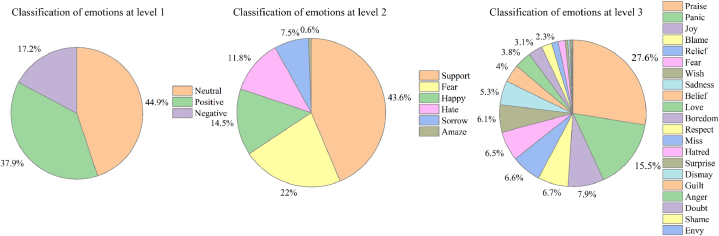
Classification of emotions at various levels during the impact of Typhoon Doksuri.

By combining the features of the “Doksuri” typhoon disaster with the Chinese emotion vocabulary ontology library created by the DUTIR as the foundational lexicon, a specialized lexicon of sentiment related to the typhoon was created. First, the microblog text was matched with the emotion dictionary to extract 204,086 emotion keywords, and the extracted keywords were then multilevel categorized based on the emotion dictionary. The results are presented in Fig. 7, where the first-level emotions indicate more neutral emotions and are followed by positive emotions and relatively few negative emotions, and this indicates that the public basically maintains an optimistic attitude toward typhoon disasters. The results of the secondary emotion categorization include “Support”, “Fear”, “Happy”, “Hate”, “Sorrow”, “Amaze”, and supportive emotions accounted for the highest percentage at 43.6 %. This was followed by “Fear” at 22 %, then “Happy” and “Hate” at a higher percentage, and finally “Sorrow” and “Amazed” at a lower percentage. The results of the three-level categorization of emotions include “Praise”, “Panic”, “Joy”, “Blame”, “Relief”, “Fear”, “Wish”, “Sadness”, “Belief”, “Love”, “Boredom”, “Respect”, “Miss”, “Hatred”, “Surprise”, “Dismay”, “Guilt”, “Anger”, “Doubt”, “Shame”, “Envy”, and “Praise” exhibits the highest proportion of emotions, “Panic” is the second highest, and the proportion of emotions such as “Joy”, “Blame”, “Relief”, “Fear”, and “Wish” remains relatively unchanged.

**Fig. 8:**
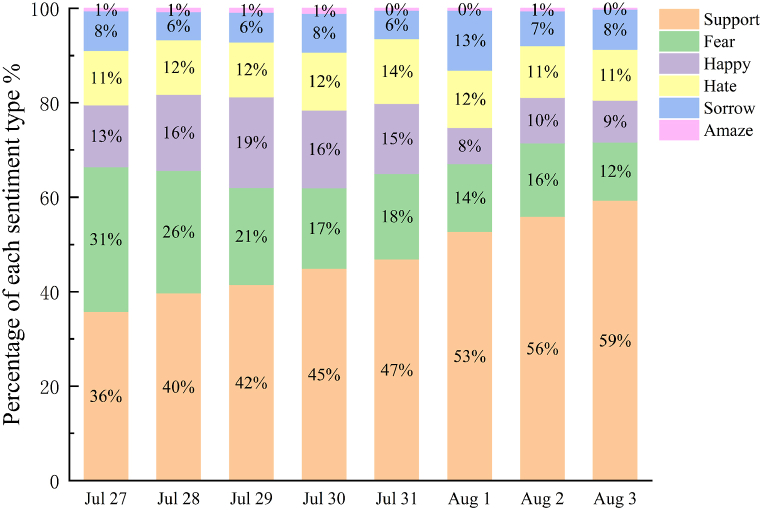
Percentage of daily mood types during the impact of Typhoon Doksuri.

Daily sentiment classification of microblog texts was conducted during this period as presented in Fig. 8. The daily emotion classification results follow the general trend of emotion classification, with “Support” being the highest emotion type. This is followed by “Fear” and “Support” that increased gradually between July 27 and August 3, with “Support” accounting for only 36 % of the emotion types on July 27 and rising to 59 % of the emotion types on August 3. In contrast, the proportion of the emotion “Fear” decreased daily. The emotion “Fear” was 31 % on July 27, and on August 3, the emotion “Fear” dropped to 12 %. Other emotions such as “Happy”, “Hate”, “Sorrow”, and “Amaze” exhibited small fluctuations.

The results of sentiment classification reveals that despite a certain degree of fear and negative sentiment towards the Typhoon Doksuri disaster, the public still maintained trust in officials and expressed high praise for the rescue teams. This reflects that the public was positive regarding the measures taken by the government during the pre-disaster warning, official emergency response, rescue and relief, and post-disaster recovery periods and that public sentiment gradually changed as rescue and recovery work progressed.

### 3.4 Typical case of Mentougou Rainstorm rainfall in July of 2023

**Fig. 9:**
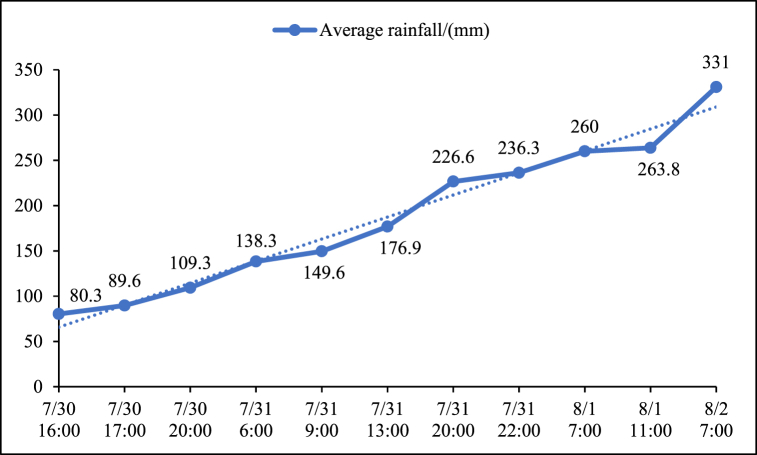
Average citywide rainfall in Beijing.

**Fig. 10:**
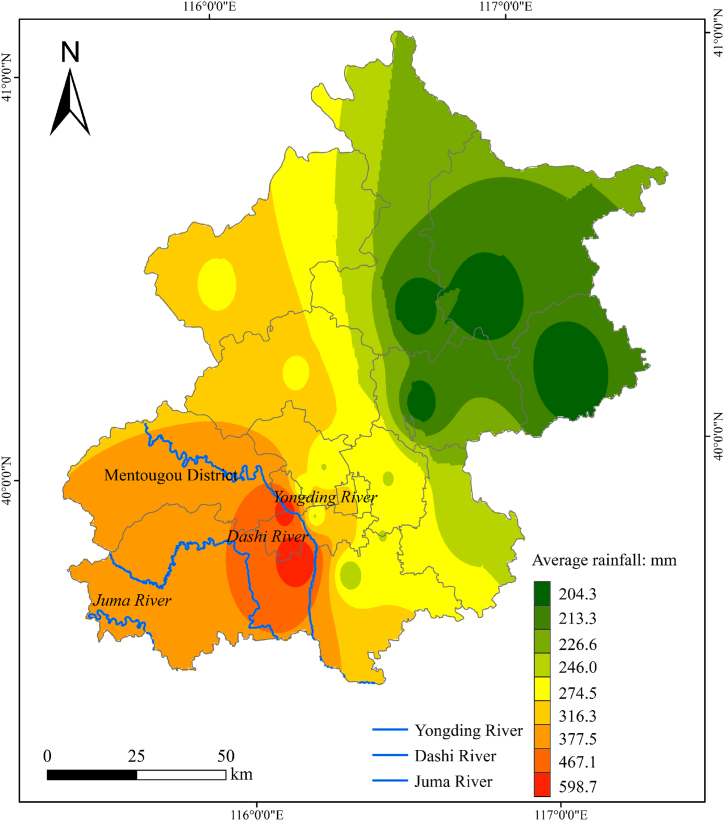
Spatial distribution of average rainfall in Beijing from July 29 to August 2, 2023.

Due to the impact of Typhoon Doksuri, from 20:00 on July 29 to 07:00 on August 2, North China, centered in Beijing, Tianjin and Hebei, suffered a historically rare exceptionally heavy rainfall. It caused more than 5 million individuals in the Beijing-Tianjin-Hebei region, with more than 1.8 million individuals being evacuated, ultimately resulting in 63 deaths and 34 missing persons. In Beijing area, rain fall continuously for 83 h, the average rainfall from 20:00 on July 29th to 16:00 on July 30th was 80.3 mm, and by 07:00 on August 2nd, the average rainfall in Beijing reached 331 mm. This was a large increase of approximately four-fold (Fig. 9). The Mentougou District averaging 538.1 mm and Fangshan District averaging 598.7 mm (Fig. 10) [27]. The floods caused by rainfall are fast, large in magnitude, and possess high peak values. The Yongding, Juma, and Dashi river basins experienced multiple rises, and the maximum flood flows were among the highest recorded in history.

Throughout the process, Mentougou and Fangshan districts received the heaviest rainfall and suffered the most serious damage. Mentougou District is located in the transition zone from the North China Plain to the Mongolian Plateau, with the terrain high in the northwest and low in the southeast. It has the highest proportion of mountains, accounting for 98.5 % of the district, and is an ecological conservation area for the capital, Beijing. The extraordinarily heavy rainfall has caused serious damage to Beijing's Mentougou district. Flash floods occurred in many areas, mudslides occurred in some areas, bridges collapsed, and over 2700 passengers on three trains were trapped for 72 h.

In summary, under the influence of Typhoon Doksuri, this historically rare extraordinarily heavy rainstorm disaster has brought great losses and impacts to the Beijing-Tianjin-Hebei region. This disaster has exerted a certain influence on the post-disaster reconstruction efforts, economic development, and social stability in the region.

### 3.5 Evaluation of classification accuracy

The results of topic categorization and the results of fine-grained secondary and tertiary sentiment categorization were evaluated for accuracy. The precision of the sentiment-analysis results for disaster events was assessed using the F1 values of the comprehensive evaluation index, Precision, and Recall. 10 % of the data was selected for topic categorization and 20 % for sentiment categorization for precision evaluation. As presented in Table 4, the Precision, Recall, and F1 values of each category are high, thus reflecting the credibility and accuracy of the public opinion topic classification results and indicating that the prediction results of the sentiment analysis model coincide with the actual emotional expressions of the public.

**Table 4:** Evaluation of classification results (%).

Categories	Precision	Recall	F1
Topic			
Typhoon Trajectory	89.56	96.34	92.83
Alert Notifications	89.02	85.38	87.16
Emergency Response	87.43	83.91	85.63
Rescue and Relief	82.71	83.97	83.33
Disaster Information	95.97	84.40	89.81
Emotion Level 2			
Support	93.19	94.50	93.84
Fear	97.28	95.70	96.48
Happy	81.52	82.20	81.86
Hate	83.63	90.32	86.85
Sorrow	95.62	89.42	92.42
Amaze	96.67	81.73	88.57
Emotion Level 3			
Praise	92.34	97.36	94.78
Panic	95.78	95.53	95.66
Joy	91.90	90.49	91.19
Blame	92.72	95.67	94.17
Relief	93.25	89.83	91.51
Fear	93.88	96.94	95.39
Wish	95.18	89.34	92.17
Sadness	94.08	95.63	94.85
Belief	94.56	81.47	87.53
Love	92.91	94.18	93.54
Boredom	96.02	88.89	92.32
Respect	90.34	92.63	91.47
Miss	95.42	94.24	94.83
Hatred	92.95	86.79	89.76
Surprise	94.09	78.67	85.69
Dismay	94.22	90.92	92.54
Guilt	91.85	93.04	92.44
Anger	91.04	94.88	92.92
Doubt	85.12	73.87	79.10
Shame	82.61	90.48	86.36
Envy	85.71	75.00	80.00

## 4 Discussion

### 4.1 Analysis of temporal and spatial changes in topics

As the typhoon route varied, the topic of dispersion yielded distinct patterns. The word frequency visualization results indicate the preparation, response, recovery, and the focus of public attention in the secondary disaster phases, and also reveal the public's knowledge and attitudes towards the disaster. The discussion of each topic peaked on the day the typhoon made landfall, after which its wind power gradually weakened, the damage caused by the typhoon diminished, the hotness of the discussion then declined. Primarily due to the observation that only major disasters that caused huge property damage or affected a large number of individuals attracted a wide range of attention in society. For example, the 2004 Indian Ocean tsunami disaster triggered a wide range of reports and fundraising activities due to it affecting a large number of Western tourist lives, and thus, it aroused global attention [28]. After Typhoon left, the topic discussion dropped rapidly and leveled off, but its residual circulation continued to move northward, thus affecting northern China. Therefore, with the exception of the topic "Typhoon Trajectory” that dropped to its lowest point and stopped fluctuating, all other topics fluctuated in line with the pattern of the daily behavior of the public, with the topic “Rescue and Relief” fluctuating the most. This was followed by “Emergency Response” and revealed that the public's tendency to discuss the topic changes according to the disaster situation. In the face of disasters, attention is often focused on the impact of the disaster itself. However, as secondary disasters unfold, the public's focus gradually shifts towards a deeper exploration of the efficacy of official government response measures and rescue deployments.

Public opinion topics were primarily concentrated in coastal areas such asFujian, Zhejiang, Guangdong that are in line with the landfall pattern of typhoons in China [29]. Coastal cities, as the landfall locations of typhoons, are the first to be affected by the impacts and damage of typhoons. Thus, the public in coastal areas exhibit a higher level of concern. In addition to coastal areas, the Beijing-Tianjin-Hebei region is at the center of the distribution of public opinion topics, weaker response to topics in other inland areas, as residual circulation of Typhoon Doksuri continued to move northward and cause catastrophic disasters in the area. Another significant factor is the higher registration rate of microblog users in the Beijing-Tianjin-Hebei region and coastal regions such as Guangdong, Fujian, and Zhejiang [30], and this leads to a much higher density of topic discussions in these regions than in other regions.

Different disaster-stricken regions exhibits variances in the categories and proportions of public topics. During the period of typhoon landfall, the primary affected areas transitioned from coastal regions progressively towards inland territories, shifted to the North China represented by the Beijing-Tianjin-Hebei region, finally extending to the Northeast. The public topics of concern in disaster-stricken areas evolved from descriptive topics such as disaster information to emergency response measures across different regions. At the onset of the disaster's impact, both officials and the public may have been underprepared or underestimated the magnitude of the disaster, resulting in a lesser focus on topics such as “Emergency Response” and “Rescue and Relief”. However, in subsequent disaster-stricken areas, there was a noticeable increase in attention towards such topics. Overall, although the suddenness and uncertainty of the disaster posed a challenge to the emergency response, the response speed was at a high level. Extensive response measures including mass evacuation, firefighting and rescue operations, disaster relief efforts, reservoir flood discharge, and emergency funding were implemented, which effectively mitigated the damage caused by the disaster.

### 4.2 Analysis of public sentiment

The evolution of public sentiment is influenced by changes in the disaster situation. During the initial phase of the disaster, a higher degree of fear is manifested by the public, particularly before and during the landfall of the typhoon. Over time and as the disaster unfolds, timely and effective measures are implemented by official and rescue teams, yielding certain degrees of success in alleviating public fear, thereby gradually shifting towards stability and positivity. As rescue efforts are gradually unfolded and achievements are attained, support for the government's response to the disaster and commendations for the rescue teams are expressed by the public, with supportive sentiments becoming the predominant emotions among the public during the disaster impact period. This reflects the public's emotional adjustment and response to the evolution of the disaster and the different stages of the official rescue work.

### 4.3 Analysis of critical incident correlation

In the Mentougou District, the river and lake levels in the entire region rose and caused a number of dangerous situations, flash floods, torrential flash floods, and urban flooding occurred in the Mentougou area, a large number of cars were washed away by the flood water, and caused great losses of life and threatened property safety. Therefore, public opinion explored the discussion of the topic during the secondary disaster impact phase in addition to the regular preparation, response, and recovery phases. Words such as “Beijing-Tianjin-Hebei” and “Mentougou” were frequently mentioned. As Mentougou is located in a mountainous area, trains scheduled for the district were also stopped due to line closure, and this caused passengers to be stranded for a long period. This aroused widespread public concern, and the related disaster buzzwords and topic discussions were at a high hotness. The primary reason why various topics are still being discussed, even after the typhoon has ceased to be cataloged, is also the continued interest of the public in the follow-up to the Mentougou extraordinarily heavy rainfall event in Beijing. The result is that in the spatial distribution of topics, the main high-heat area is the Beijing-Tianjin-Hebei region, in addition to the coastal area.

### 4.4 Recommendations for flooding risk reduction

First, strengthening urban flood control and drainage capacity. The government should strengthen infrastructure development and invest more resources in improving infrastructure such as drainage, power supply, and communication systems to enhance their disaster-resistant capacity. They should also improve water conservancy engineering facilities to enhance the disaster prevention and mitigation capacity of each facility and minimize the occurrence of basin overflows.

Second, raising public awareness of emergency responses. Before the onset of a disaster, public education on disaster prevention awareness should be strengthened, the transmission of early warning information should be accelerated, and there should be enough time for the public to respond in a timely manner so that they can take appropriate countermeasures, including stocking supplies in advance and changing travel plans.

Third, increasing attention to economically underdeveloped areas. Disasters occur more frequently in economically disadvantaged regions, as these places possess limited transportation and communication and are underdeveloped, thus prompting younger individuals to seek work elsewhere. Consequently, the population left behind is predominantly the elderly, the infirm, women, and children, ultimately leading to higher casualties during disasters. Emergency management must prioritize the assessment and response to the specific vulnerabilities of these regions by implementing timely relief measures.

Fourth, monitoring secondary disaster situations closely. Emergency management agencies and the public can maintain disaster lag awareness and be alert to possible secondary disasters while monitoring the path of the typhoon and keeping in mind that typhoons possess the typical characteristics of disaster chains [31]. There is an urgent need for relevant management authorities to strengthen the emergency response capacity for secondary disasters, enhance disaster early warning capacity, and strengthen the management of the disaster chain to improve the prevention and mitigation of typhoon disasters.

Fifth, promoting the relocation of residents in high flood risk areas. For high-risk areas such as flash flood prone areas, thorough planning should be conducted, and residents should be actively mobilized and relocated in an orderly manner.

Sixth, enhancing the collection and utilization of tweet location information. The number of tweets containing latitude and longitude information in the microblog data collected during Typhoon Doksuri was much smaller than the number of microblog texts obtained. A greater amount of user location information during disaster management is more helpful for the emergency management department or rescue teams for allowing them to quickly locate the positions of the affected individuals so that disaster relief resources can respond quickly to meet the different needs of disaster relief in various locations that may have different disaster relief requirements. Finally, emergency management departments and official governments can respond quickly to the public opinion topics and emotional tendencies of users and adjust relevant emergency measures and rescue operations to respond to disasters more effectively and protect public safety.

### 4.5 Limitations and future work

The spatiotemporal distribution and emotional tendencies of public discourse during typhoon disaster events are explored, however, it still has limitations. In the public opinion analysis stage, the analysis was only conducted at the provincial administrative scale without further extracting the geographic location information contained in the text for a finer-grained analysis. The constraint may lead to insufficient understanding of the performance and influence of public opinion topics at a finer scale. Future research could achieve a deeper exploration of geographic location information through more refined geographic information extraction techniques, such as toponym recognition. There are also limitations in the choice of analysis algorithms, in the future, we will combine more data (e.g., long temporal or large spatial study) and more efficient training algorithms and architectures, such as Bert, Transformer, GPT, etc [[32], [33], [34], [35]], to further deepen the research and application promotion.

## 5 Conclusion

Based on the microblog text data of the “Doksuri” typhoon disaster, this study conducts a multidimensional analysis of the hot topics during this disaster event, explore differences in topic response and distribution between coastal and inland areas. It also employs a more in-depth and granular approach to explore the emotional tendencies of the public and changes during a disaster. Additionally, the Mentougou extraordinarily heavy rainstorm is used as a typical case to explore the temporal and spatial changes related to the distribution of public opinion topics. During an emergency event, the focal points of public attention shift with the movement of the typhoon path and the passage of time. The distribution of public opinion hotness correlates with the impact scope of the “Doksuri” Typhoon disaster, population density, and economic development levels. The time series of various topics exhibited synchronywith the typhoon wind power changes. Topics such as “Disaster Information” and “Alert Notifications” have garnered significant public attention. Geospatially, public opinion on each topic is centered on coastal areas and the Beijing-Tianjin-Hebei region. Coastal areas, due to unique geographic location, are more responsive and quicker in terms of topic response, compared to inland areas where topic response is slower. During the “Doksuri” typhoon disaster, the prevailing sentiment is predominantly “Support” that gradually increased over time, while the sentiment of “Fear” diminishes gradually. During heavy rains in Mentougou (ranking among the top districts in Beijing), the amount of rainfall gradually increased and caused flooding and overflow of the watershed. Based on this, referenceable countermeasures are proposed for both the public and the authorities to enhance public awareness of risk prevention, promote the soundness of the emergency management system, enhance the disaster prevention, mitigation and relief capacity.

## Funding

This work was funded by the National Key R&D Program of China (Grant No. 2023YFE0208000), and the Construction Project of 10.13039/501100012455China Knowledge Centre for Engineering Sciences and Technology (Grant No. CKCEST-2023-1-5).

## Data availability statement

Data included in article/supp. Material/referenced in article.

## CRediT authorship contribution statement

**Mengqiong Xu:** Writing – review & editing, Writing – original draft, Visualization. **Juanle Wang:** Writing – review & editing, Supervision, Methodology. **Zheng Qu:** Resources. **Xiaodong Min:** Investigation. **Yamin Sun:** Investigation.

## Declaration of competing interest

The authors declare the following financial interests/personal relationships which may be considered as potential competing interests:Juanle Wang reports financial support was provided by the National Key R&D Program of China. If there are other authors, they declare that they have no known competing financial interests or personal relationships that could have appeared to influence the work reported in this paper.
